# Une forme exceptionnelle de la luxation perilunaire du carpe

**DOI:** 10.11604/pamj.2014.18.108.4439

**Published:** 2014-06-03

**Authors:** Issam Elouakili, Younes Ouchrif, Abdeljaouad Najib, Redouane Ouakrim, Omar Lamrani, Mohammed Kharmaz, Farid Ismael, Abdo Lahlou, Mohammed Elouadghiri, Ahmed El Bardouni, Mustapha Mahfoud, Mohammed Saleh Berrada, Mouradh El Yaccoubi

**Affiliations:** 1 Service de Traumatologie-Orthopédie, CHU Ibn Sina, Rabat, Maroc

**Keywords:** Poignet, luxations périlunaires, nécrose du semilunaire, wrist, Perilunar dislocations, necrosis of the lunate

## Abstract

Les luxations périlunaires (LPL) du carpe sont des lésions extrêmement rares, qui peuvent passer inaperçue en raison d'un tableau clinique souvent trompeur, des radiographies en profil non strict ou d'interprétation difficile. Nous rapportons l'observation d'une luxation périlunaire stade III selon la classification de Witvoët et Allieu chez un patient de 32 ans, il s'agit d'une forme encore plus rare voire exceptionnelle et qui peut induire de sérieux problèmes en raison de la sévérité des dommages ligamentaires et du risque de nécrose du semilunaire plus important dans ce type de lésions. Le traitement est toujours chirurgical et doit être réalisé dans les plus brefs délais afin d’éviter les complications.

## Introduction

Les luxations périlunaires du carpe sont des lésions extrêmement rares, qui peuvent passer inaperçues dans 15 à 50% des cas [1, 2]. Elles résultent généralement d'un choc violent dans le cadre d'un traumatisme à haute énergie. Elles sont responsables de lésions ostéo-cartilagineuses et capsulo-ligamentaires graves, qui peuvent laisser des séquelles fonctionnelles Importantes, dominées par l'instabilité chronique du poignet et à long terme par l'arthrose [3–5]. Les auteurs rapportent une forme anatomique exceptionnelle de luxation postérieure du carpe, liée à un déplacement proximal et isole du lunatum stade III de Witvoët et Allieu [2] traité chirurgicalement avec résultat fonctionnel satisfaisant à 12 mois de recul.

## Patient et observation

**Figure 1:**
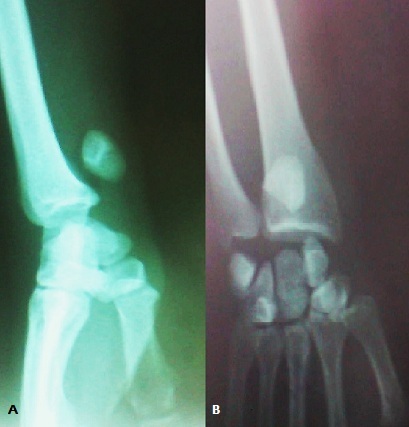
Aspect radiologique de face (A) et de profil (B) de la luxation perilunaire stade III du carpe

**Figure 2:**
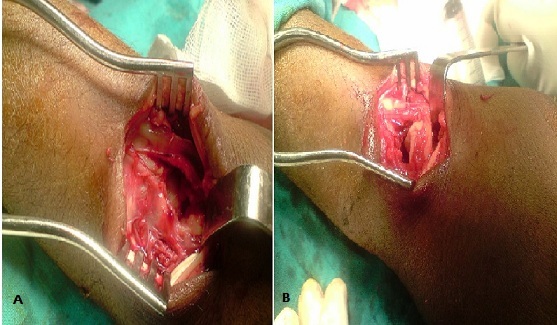
Image per opératoire A) emplacement du semilunaire entre radius et hamatum; B) remise du semilunaire libre de toutes attaches dans son emplacement

**Figure 3:**
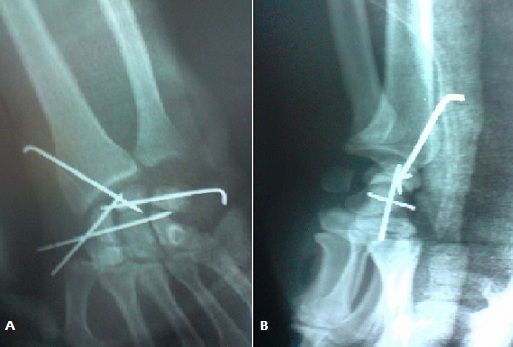
Aspect radiologique en post operatoire de face (A) et de profil (B)

**Figure 4:**
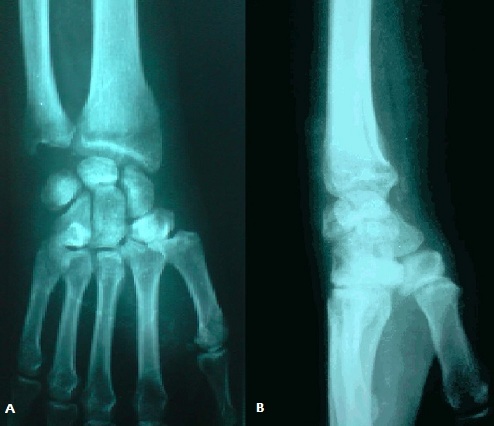
Aspect radiologique de face (A) et de profil (B) à 6 mois de recul

M. L.A, âgé de 32 ans cycliste professionnel, sans antécédents, a présenté suite à une chute de son vélo, un traumatisme du poignet droit associé à un traumatisme cranio faciale. L'examen à l'admission a trouvé un patient conscient stable avec oedème et déformation du poignet droit, sans ouverture cutanée, ni lésions vasculaires ou nerveuses, notamment pas d'atteinte du nerf médian. Toute mobilisation du poignet droit était impossible et douloureuse. Le bilan radiologique réalisé a objectivé une luxation périlunaire postérieure du carpe avec avulsion proximale du lunatum situé en regard de l’épiphyse distale du radius, sans fracture de l'os scaphoïde (Figure 1). Une intervention chirurgicale en urgence a été pratiquée par voie postérieure (Figure 2) dont l'exploration a trouvé une rupture du ligament annulaire antérieur du carpe, des freins ligamentaires antérieur et postérieur du lunatum ainsi que des ligaments scapholunaire et triquetrolunaire, laissant le lunatum libre sans aucune attache. Le patient a ainsi bénéficié d'une réduction du lunatum avec fixation du carpe par embrochage scapholunaire, lunotriquetral, scaphohamatal et radiolunaire (Figure 3). Une immobilisation plâtrée complémentaire par une attelle intrinsèque a été réalisée pendant six semaines au bout desquelles les broches ont été enlevées et une rééducation a été entreprise. Au recul de six mois le patient ne présentait pas de douleur résiduelle et sa mobilité était de 45° en flexion, 70° en extension, 25° en inclinaison radiale, 40° en inclinaison cubitale, 90° en pronation et 90° en supination. Sa prise de force, comparée au côté sain, était presque normale. Sur le plan radiologique, on note une condensation avec une légère bascule en VISI (Figure 4).

## Discussion

Les luxations périlunaires du carpe sont rares et constituent 5 à 10% des lésions traumatiques du poignet [6]. La fréquence élevée des retards diagnostiques observés est due à un tableau clinique souvent trompeur, des radiographies en profil non strict ou d'interprétation difficile. Un polytraumatisme associé peut aussi masquer le problème carpien. Il s'agit d'un tiers des cas selon Witvoët et allieu [2] et 25% des cas selon Herzberg [1]. Le mécanisme physiopathologique de la luxation périlunaire postérieure est une chute sur un poignet en hyperextension et en inclinaison ulnaire et c'est l'angle d'hyperextension qui va déterminer la sévérité de la luxation [7].

Dans notre observation le patient a présenté une luxation périlunaire stade III selon la classification de Witvoët et Allieu. Il s'agit d'une entité encore plus rare voire exceptionnelle, dont le pourcentage est variable selon les séries, ainsi Herzberg [1] n'a trouvé aucun cas dans une étude multicentrique de 166 cas de luxation périlunaire alors que Lacour [8] a trouvé quatre cas dans une série de 62 patients. Ce type de luxation expose à un plus grand risque de nécrose du semilunaire, ceci s'explique par le faite que la vascularisation de ce dernier est assurée dans sa quasi-totalité par le frein antérieur [9] qui se trouve rompue en cas de luxation stade III alors qu'il reste intact dans les stades I et II, ainsi Lesire [10] dans sa série de 110 cas dont 12 de stade III n'a trouvé aucun cas de nécrose en cas de stade I, 16,6% dans le stade II et 50% dans le stade III. Le dé1ai d′apparition des nécroses varie entre un mois et 12 ans après le traumatisme [10] cependant à ne pas confondre avec une hypercondensation transitoire du lunatum à la radiographie, celle-ci pouvant persister encore quelques mois après un traitement correct chose qu'on a observé chez notre malade sur la radio de contrôle faite à 12 mois de recul (fig 4). Autres facteurs peuvent aussi influencer le pronostic de ce type de lésion, on note ainsi le délai de la prise en charge. Dans la série de Lesire [10] quatre cas ont été diagnostiqués tardivement dont deux ont bénéficié d'une lunarectomie d'emblée et les deux autres ont évolués vers la nécrose secondairement. L'autre facteur qui a été signalé par Lesire est l'association d'une voie d'abord antérieure et une voie postérieure.

Le traitement chirurgical des LPL stade III rejoint celui des autres types mais avec quelques particularités. Trois types de traitement ont été décrits: la réduction fermée et immobilisation plâtrée, la réduction fermée et embrochage percutané associés à une contention rigide, et enfin, la réparation ligamentaire et osseuse par voie ouverte associée également à une contention rigide. Actuellement, la réduction orthopédique non sanglante donne des résultats non satisfaisants [11] ne permettant pas des gestes complémentaires sur l'appareil capsulo-ligamentaire et donc sur la stabilité du carpe. Un embrochage percutané lunoscaphoidien et lunotriquetral peut être réalisé sous contrôle de l'amplificateur de brillance. Cependant, l'embrochage percutané est difficile et sa supériorité n'est pas prouvée [12]. La réparation chirurgicale par une voie d'abord antérieure est logiquement plus intéressante permettant de réaliser des gestes de réparations ligamentaires mais une voie postérieure peut également être utilisée comme c’était le cas chez notre patient [7], une double voie d'abord doit être évitée dans les stades III. La lunarectomie, la résection de la première rangée ou l′arthrodèse du poignet ne doivent être réservées qu′aux échecs du traitement réparateur ou en cas de LPL stade III vue tardivement. Plusieurs facteurs de mauvais pronostic ont été retenus par les auteurs ainsi Garcias ‘Elias et al [13], insistent, en premier lieu sur le délai entre l'accident et la réduction puis la précision de la réduction et le degré de bascule du semilunaire, Witvôet et Allieu ont aussi insisté sur la bascule du semilunaire et la rupture des freins de ce derniers. En fait toutes ces constatations restent très subjectives et on n'a trouvé aucune étude qui s'est intéressée à suivre l’évolution de ces lésions stade III pour bien définir les facteurs de mauvais pronostic et rétablir ainsi une stratégie bien adaptée de prise en charge thérapeutique.

## Conclusion

La luxation perilunaire du carpe stade III est une forme exceptionnelle qui nécessitera une prise en charge thérapeutique précoce et bien adaptée, et un suivi régulier afin de remédier aux complications fréquentes observées dans ce type de lésions notamment la nécrose du semilunaire.
